# A US–CHINA COMPARISON ON THE DETERMINANTS OF RESIDENTIAL RELOCATION AND ASSOCIATED MORTALITY RISK

**DOI:** 10.1093/geroni/igae098.2345

**Published:** 2024-12-31

**Authors:** Dexia Kong, Peiyi Lu

**Affiliations:** The Chinese University of Hong Kong, Hong Kong, China (People’s Republic); The University of Hong Kong, Hong Kong, China (People’s Republic)

## Abstract

Many middle-aged and older adults relocate to pursue a better living environment. However, existing studies have inconclusive evidence on the determinants of relocation and its health implications, as well as lack a cross-national perspective. This study sought to compare the US and China in examining the determinants of residential relocation and its associated mortality risk. Longitudinal data from the US Health and Retirement Study in 2010-2018 and from China Health and Retirement Longitudinal Study in 2011-2018 were used (NUS=20,292, NChina=11,694). Community-dwelling respondents (aged 50+) reported whether they had relocated and were followed up until 2018. Log-binomial regression model investigated the significant predictors of relocation. Cox survival analysis examined the relationship between relocation and all-cause mortality, adjusting for baseline covariates and accounting for selection bias. About 19% American and 17% Chinese respondents relocated between communities over the study period. The common positive predictors of relocation in both countries were younger age, higher education, urban residence, and renting (relative risk>1, P< 0.05). Relocation was associated with lower mortality risk in both countries (US: HR=0.63, 95%CI=0.57, 0.70; China: HR=0.40, 95%CI=0.32, 0.50), and this association was significantly stronger in China compared to the US. (Prelocation×country=0.0009). The determinants of relocation in the US and China showed similarities, aligning with the person-environment framework. Residential relocation was linked to increased survival advantages in both countries, probably because old adults who relocated can make informed and voluntary decisions to relocate and adjust effectively following the move. Pre- and post-relocation interventions are suggested to facilitate the process of moving.

